# Supplementary material for: Comparative Histopathological and Morphometric Analysis of Lung Tissues in Stillborn Cubs of South China Tiger and Amur Tiger
Source: Biology (Basel). 2025 Jul 8;14(7):833. doi: 10.3390/biology14070833 (PMC12292684; doi:10.3390/biology14070833)
Supplement: Supplementary file 1 [file biology-14-00833-s001.zip › biology-3628358-supplementary.pdf]

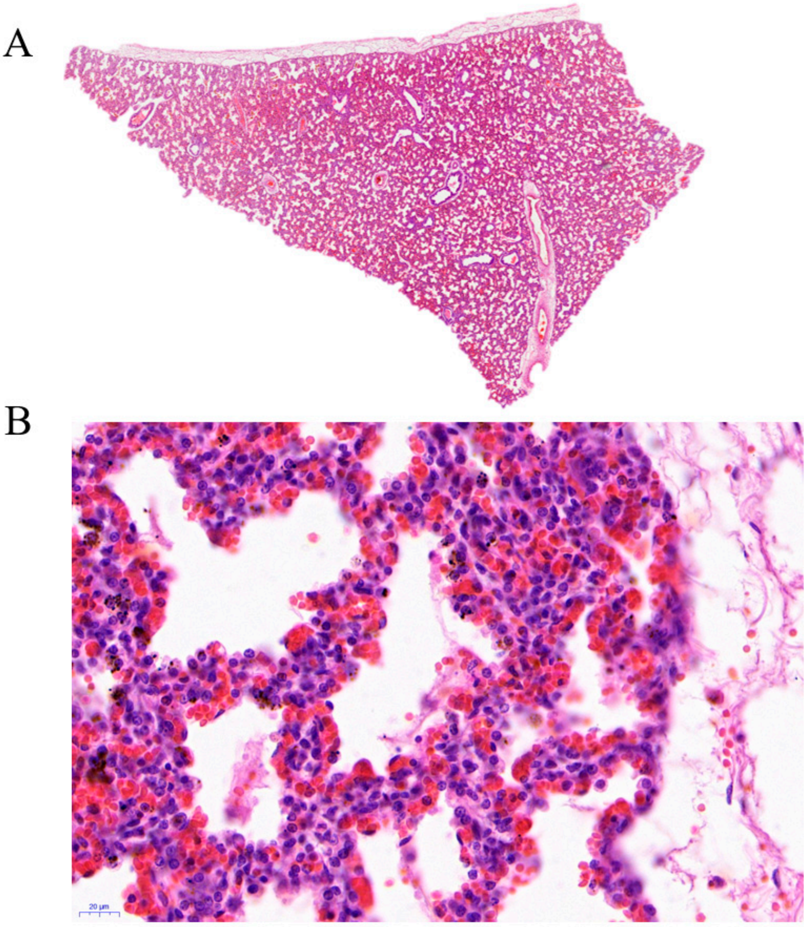

Figure S1. H&E-stained lung tissue of SCT_stb1. A represents the low-magnification field of view, while B corresponds to the high-magnification field of view.

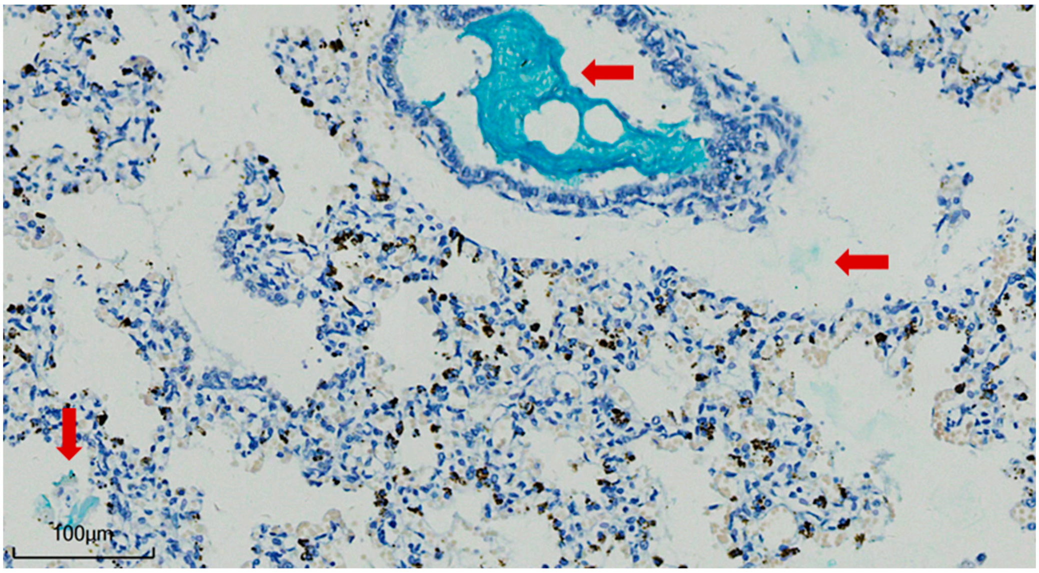

Figure S2. Alcian blue-stained lung tissue of SCT_stb1. The location of the red arrow in the image indicates the composition of meconium.

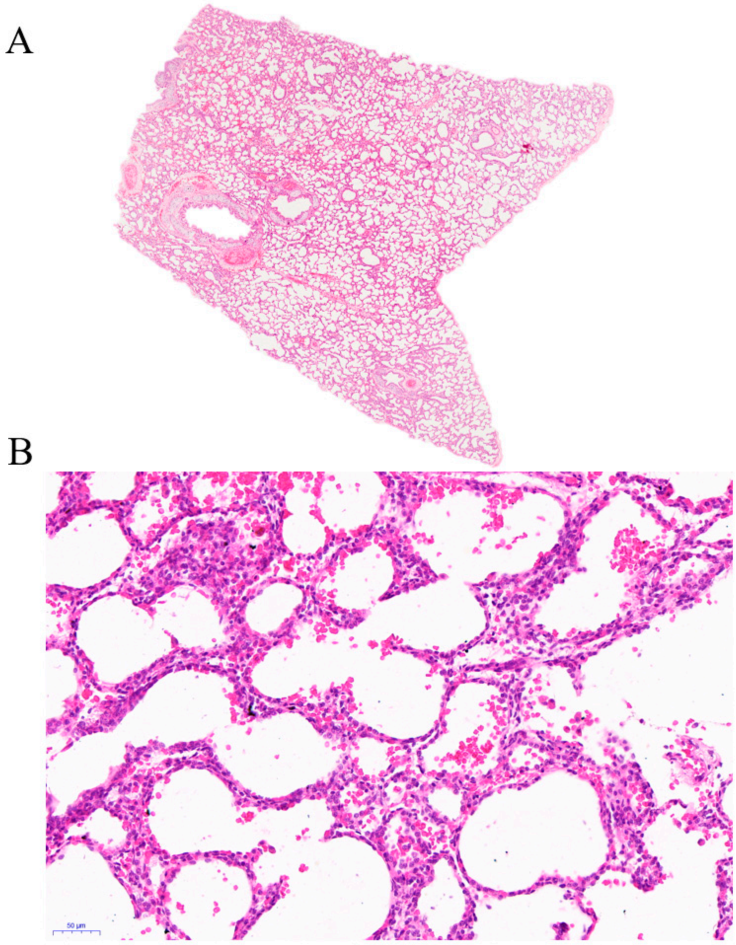

Figure S3. H&E-stained lung tissue of SCT_stb2. A represents the low-magnification field of view, while B corresponds to the high-magnification field of view.

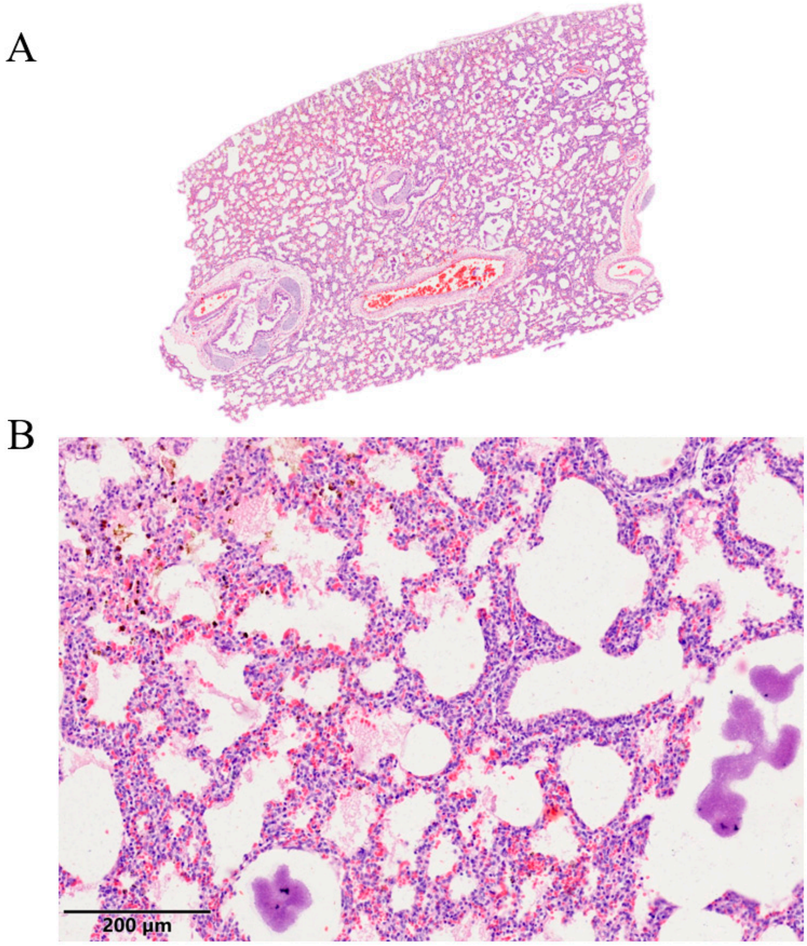

Figure S4. H&E-stained lung tissue of SCT_alv. A represents the low-magnification field of view, while B corresponds to the high-magnification field of view.

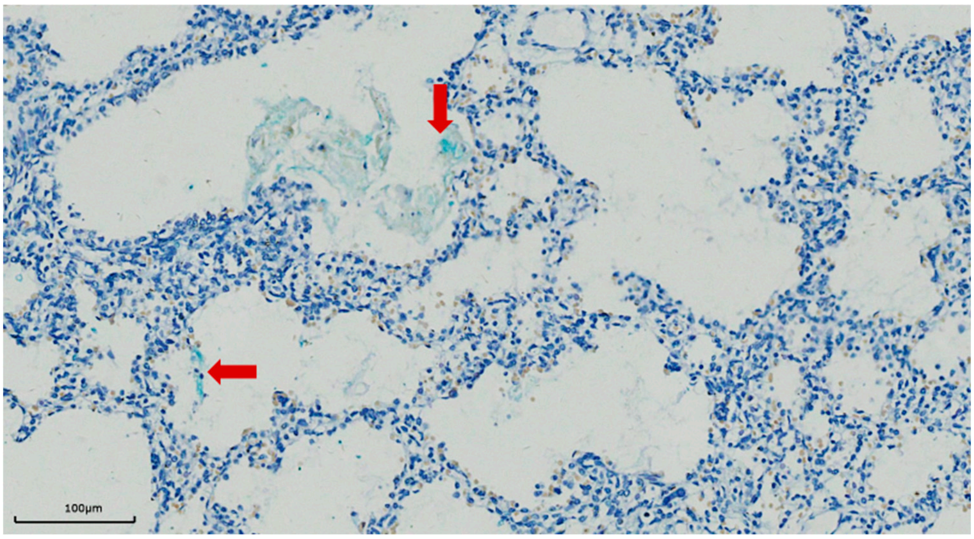

Figure S5. Alcian blue-stained lung tissue of SCT_alv. The location of the red arrow in the picture indicates the composition of meconium.

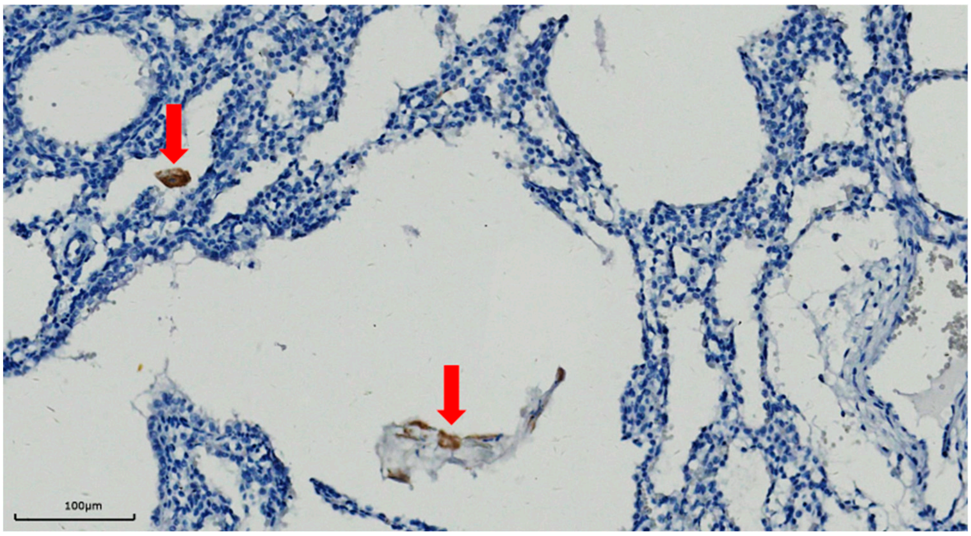

Figure S6. Immunohistochemically stained lung tissue of SCT_alv. The red arrows indicate positively stained keratin.

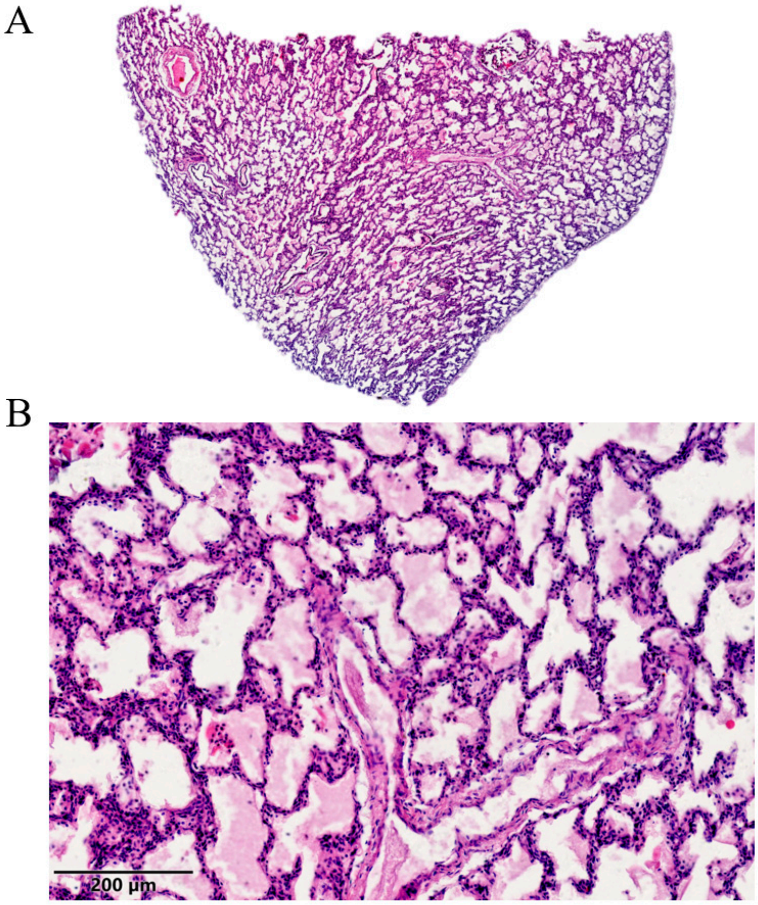

Figure S7. H&E-stained lung tissue of AT_utd. A represents the low-magnification field of view, while B corresponds to the high-magnification field of view.

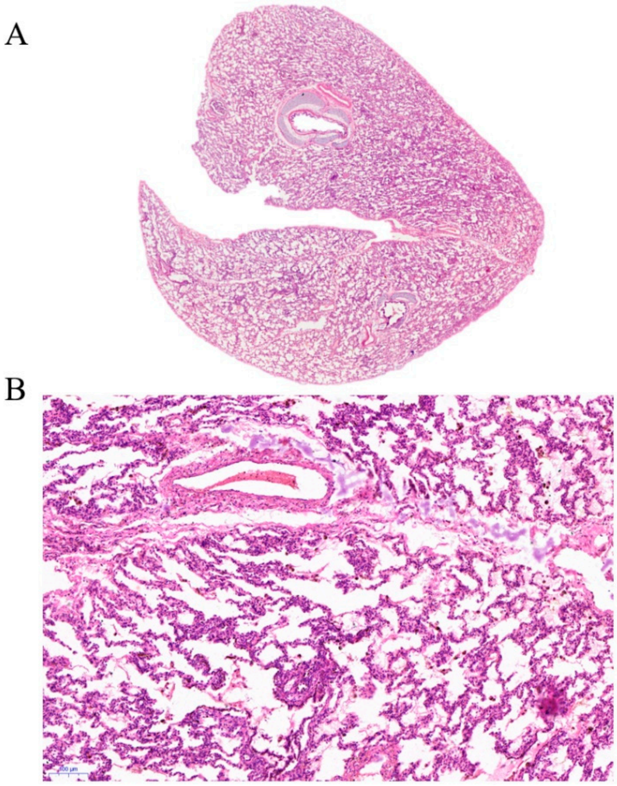

Figure S8. H&E-stained lung tissue of AT_stb1. A represents the low-magnification field of view, while B corresponds to the high-magnification field of view.

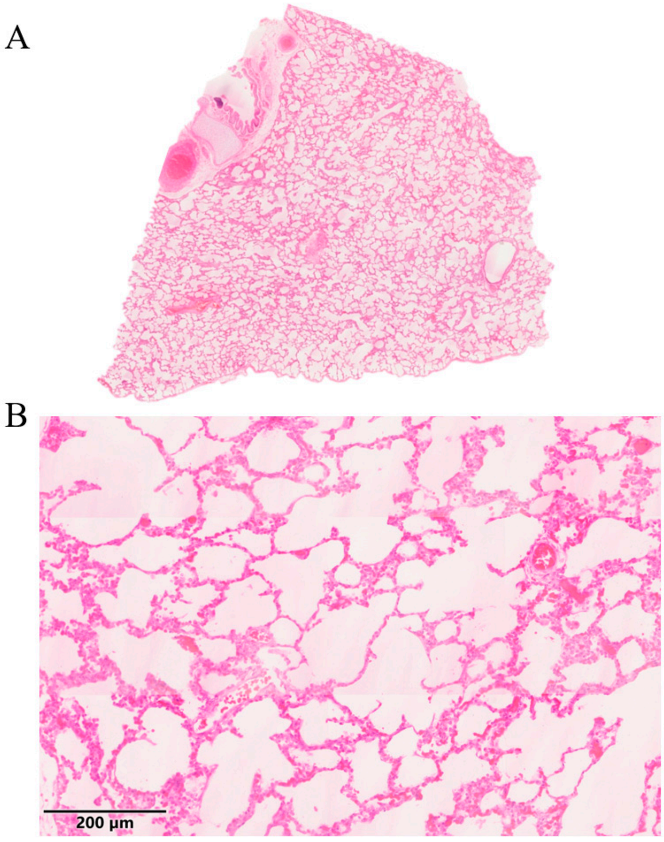

Figure S9. H&E-stained lung tissue of AT_stb2. A represents the low-magnification field of view, while B corresponds to the high-magnification field of view.
